# Deep transformer-based heterogeneous spatiotemporal graph learning for geographical traffic forecasting

**DOI:** 10.1016/j.isci.2024.110175

**Published:** 2024-06-25

**Authors:** Guangsi Shi, Linhao Luo, Yongze Song, Jing Li, Shirui Pan

**Affiliations:** 1 Department of Chemical & Biological, Faculty of Engineering, Monash University, Clayton, VIC 38000, Australia; 2 Department of Data Science & AI, Faculty of IT, Monash University, Clayton VIC 38000, Australia; 3 School of Design and the Built Environment, Curtin University, Perth, WA 6845, Australia; 4 School of ICT, Griffith University, South Port, QLD 4215, Australia

**Keywords:** Geography, Artificial intelligence, Engineering

## Abstract

Accurate geographical traffic forecasting plays a critical role in urban transportation planning, traffic management, and geospatial artificial intelligence (GeoAI). Although deep learning models have made significant progress in geographical traffic forecasting, they still face challenges in effectively capturing long-term temporal dependencies and modeling heterogeneous dynamic spatial dependencies. To address these issues, we propose a novel deep transformer-based heterogeneous spatiotemporal graph learning model for geographical traffic forecasting. Our model incorporates a temporal transformer that captures long-term temporal patterns in traffic data without simple data fusion. Furthermore, we introduce adaptive normalized graph structures within different graph layers, enabling the model to capture dynamic spatial dependencies and adapt to diverse traffic scenarios, especially for the heterogeneous relationship. We conduct comprehensive experiments and visualization on four primary public datasets and demonstrate that our model achieves state-of-the-art results in comparison to existing methods.

## Highlights

• We identify a new heterogeneous spatiotemporal relationship in traffic data
• We propose a novel method TSTGNN for geographical traffic forecasting
• Our method achieves state-of-the-art results compared with other methods

## Graphical abstract

**Figure:**
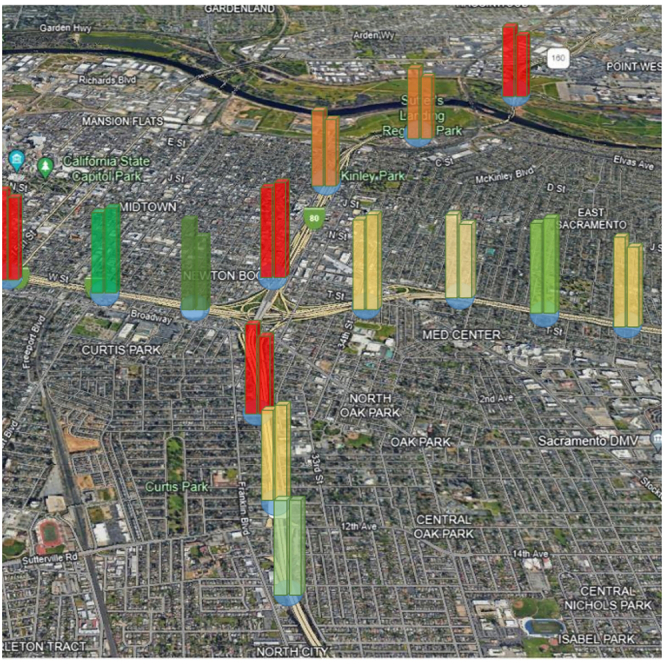

## Introduction

Building intelligent geospatial artificial intelligence (GeoAI) systems have attracted significant attention in recent years.^1^ Among the many efforts, prediction of traffic patterns is vital for urban transportation planning,^2^ traffic management,^3^^,^^4^ and intelligent transportation systems.^5^ In recent years, researchers have focused heavily on this area due to its potential to mitigate traffic congestion, optimize transportation resources, and improve public safety. Thanks to the advancement of sensor networks and location-based services, a significant amount of spatiotemporal traffic data are now available. This has enabled the development of data-driven models that can accurately predict traffic patterns using a variety of techniques to effectively capture the complex spatiotemporal dependencies inherent in traffic data, ranging from traditional statistical method^6^ to deep learning^7^ and graph-based deep learning approaches.^8^^,^^9^^,^^10^^,^^11^

Traditional methods for geographical traffic forecasting primarily rely on time series analysis and statistical models, such as autoregressive integrated moving average (ARIMA),^6^ exponential smoothing state space models,^12^ and Kalman filtering techniques.^13^ These models have been widely used due to their simplicity and interpretability. However, they often fail to capture the complex spatial dependencies inherent which is significant for geographical data,^14^ leading to suboptimal forecasting performance.

Deep learning-based methods have emerged to address the limitations of traditional models, employing techniques such as recurrent neural networks (RNNs)^15^ and convolutional neural networks (CNNs) (LSTNet).^7^ While these methods have shown improved performance over traditional methods, they still face challenges in capturing long-term temporal dependencies and accurately modeling the complex spatial relationships in geographical traffic data.

Recent advancements in deep learning, particularly graph neural network (GNN)-based models such as graph convolutional network (GCN)^16^ and graph attention network (GAT),^17^ have shown promise in overcoming limitations of both traditional and deep learning-based methods by effectively capturing spatial dependencies in geographical traffic data. For instance, ASTGCN^18^ and STG2Seq^19^ have been developed to capture spatiotemporal correlations. Meanwhile, STSGCN^20^ utilizes multiple localized spatiotemporal subgraph modules to directly capture localized spatiotemporal correlations. LSGCN^21^ integrates a novel attention mechanism and graph convolution into a spatial gated block, while AGCRN^22^ utilizes node adaptive parameter learning to automatically capture node-specific spatial and temporal correlations without a pre-defined graph. STFGNN^23^ captures hidden spatial-dependencies by fusing various spatial and temporal graphs treated for different time periods in parallel, while Z-GCNETs^24^ integrates a new time-aware zigzag topological layer into time-conditioned GCNs. STGODE^25^ captures spatiotemporal dynamics through a tensor-based ODE. StemGNN^26^ utilizes the spectral-temporal method for the multivariate time series including traffic forecasting tasks. However, these models still have limitations, including difficulty in modeling long-term dependencies by information fusion and inefficiency in capturing dynamic spatial dependencies with fixed graph structure, ignoring the potential heterogeneous relationships.

To address this issue, ASTGNN^27^ proposes an attention-based spatiotemporal GNN to capture the periodicity and the spatial heterogeneity of traffic data, and long-term forecast. ST-WA^28^ encodes time series from different locations into stochastic variables to generate location-specific and time-varying model parameters to better capture the heterogeneous spatiotemporal dynamics. Although some heterogeneous patterns are modeled, there are many forms of heterogeneous spatiotemporal graphs that need to be explored in geographical traffic forecasting such as the rate of traffic flow change, series trend, and so on. Moreover, due to the implementation of various spatiotemporal decomposition approaches for multivariable time series analysis, there is a potential risk of heterogeneity being altered or lost. This issue underscores the complexity of accurately maintaining the distinct characteristics of the data throughout the analytical process. Thus, several challenges remain in this area.

(1) Challenge 1. Capturing long-term temporal dependencies: existing deep learning methods like RNNs, CNNs, and FC-LSTM often struggle with capturing long-term patterns due to their inherent limitations. This challenge is particularly pronounced in geographical traffic forecasting, as the historical data often span long time horizons, and understanding the long-term dependencies is crucial for accurate predictions.
(2) Challenge 2. Modeling dynamic spatial dependencies: many graph-based models rely on static adjacency matrices for graph convolutions, which may not effectively capture dynamic spatial dependencies that arise due to varying geographical traffic conditions or evolving road network structures. Accurately modeling these specific dynamic spatial relationships is essential for capturing the complex interactions among geographical traffic sensors in different geographical locations.
(3) Challenge 3. Although existing spatiotemporal GNN-based methods have been successful in incorporating both temporal and spatial information into high-dimensional space to capture their dependencies, they often adopted a fixed graph when aggregating spatial information, without considering the dynamics of relationships among layer-to-layer future transformations. Particularly when we observe that the information of neighboring points in spatial locations demonstrates opposite relationships in temporal dimensions, which is called spatiotemporal heterogeneous relationship, the previous methods cannot work well. Thus, this relationship also needs to be adaptively adjusted to accommodate the change of features in the high-dimensional space to capture the spatiotemporal heterogeneous relationship.

The accelerated evolution of large language models has significantly heightened the focus on transformer-based methodologies within the realm of time series analysis.^29^ The scholarly article^30^ endeavors to encapsulate the deployment of diverse transformer-based techniques in the study of time series, thereby elucidating their formidable potential and efficacy. FEDformer^31^ proposes to combine transformer with the seasonal-trend decomposition method, in which the decomposition method captures the global profile of time series while transformers capture more detailed structures. Time-LLM^32^ presents a reprogramming framework to repurpose LLMs for general time series forecasting with the backbone language models kept intact.

In this paper, we propose a novel deep transformer-based heterogeneous spatiotemporal graph neural network (TSTGNN for abbreviation) for geographical traffic forecasting, which addresses the aforementioned challenges and offers the following contributions.

(1) Contribution 1: instead of relying on data fusion, our model employs a temporal transformer to capture the temporal dependencies in the long horizon of historical data. The transformer architecture^33^ has demonstrated superior performance in various sequence-to-sequence tasks due to its ability to model long-term dependencies and parallelization capabilities. By leveraging the transformer’s self-attention mechanism, our model can effectively capture complex temporal patterns in geographical traffic data without the need for data fusion.
(2) Contribution 2: to handle complicated spatial dependencies, we propose an adaptive normalized graph structure in different graph convolution layers. Unlike previous works that use a fixed graph structure, our approach allows the graph structure to vary as the layer changes and effectively captures dynamic spatial dependencies. Moreover, a novel layer normalization can adaptively learn the underlying spatiotemporal heterogeneous relationships among geographical traffic sensors layer by layer, improving the model’s ability to generalize to different geographical traffic scenarios.
(3) Contribution 3: we conduct extensive experiments on four main geographical traffic datasets: PeMSD3, PeMSD4, PeMSD7, and PeMSD8. Our model achieves state-of-the-art results compared to existing methods, demonstrating its effectiveness in addressing the aforementioned challenges. Additionally, we present ablation studies and parameter sensitivity analyses to further validate the robustness and generalizability of our model.

Our paper is organized as follows. At the beginning, we present a summary, the introduction, and the related work in geographical traffic forecasting, focusing on the challenges and limitations of existing traditional, deep learning-based, and graph-based deep learning models. The next results section describes the main results, including the overview of our method, datasets, baselines, and performance comparison and visualization on the four main geographical traffic datasets. In the discussion section, we report the experimental discussion including visualization of error, and spatiotemporal heterogeneous relationship. This section also discusses the model’s robustness and generalizability in addressing the challenges mentioned earlier. The final section illustrates the methodology of our method including temporal transformer layers, layer normalization mechanism, and graph convolution layers.

## Results

### Datasets

**Figure 1:**
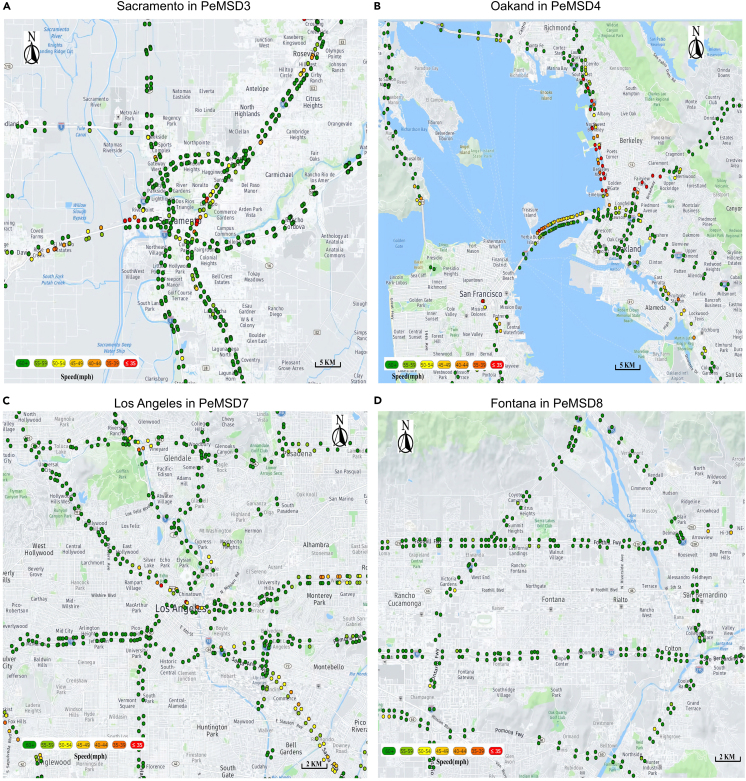
The visualization of the cites in maps with sensor installation site in these datasets (A) is Sacramento in PeMSD3, (B) is Oakland in PeMSD4, (C) is Los Angeles in PeMSD7, and (D) is Fontana in PeMSD8.

In this section, we briefly introduce the open-source Caltrans Performance Measurement System (PeMS), which serves as our primary investigative focus. Database: PeMS represents a comprehensive transportation data collection and analysis system, delivering real-time geographical traffic information for California’s highways. PeMS amasses data from a diverse array of sources, encompassing geographical traffic sensors, weather stations, and incident reports. Notably, PeMSD3, PeMSD4, PeMSD7, and PeMSD8 are among the most extensively utilized datasets provided by PeMS and we visualized some partial sensor data of featured cities in this dataset such as Sacramento in PeMSD3, Oakland in PeMSD4, Los Angeles in PeMSD7, and Fontana in PeMSD8. The visualization of the dataset maps is illustrated in Figure 1. Meanwhile, we set two main tasks: single-step prediction in which one step forecasting based on the m steps historical information and multi-steps prediction in which n steps prediction based on the m steps historical information.

These datasets have garnered widespread adoption among transportation researchers, planners, and engineers, facilitating the study of geographical traffic patterns and the development of strategies to enhance transportation systems. Through real-time data collection and analysis, PeMS empowers policymakers to make informed decisions, contributing to the optimization of efficiency and safety on California’s highways. The detailed information has been summarized in Table 1.

**Table 1:** A summary of the datasets used in our work

Dataset	|V|	Time steps	Time range	Type
PeMSD3	358	26,208	09/2018–11/2018	Volume
PeMSD4	307	16,992	01/2018–02/2018	Volume
PeMSD7	883	28,224	05/2017–08/2017	Volume
PeMSD8	170	17,856	07/2016–08/2016	Volume

We predict either geographical traffic volume or velocity.

### Overview of our method

**Figure 2:**
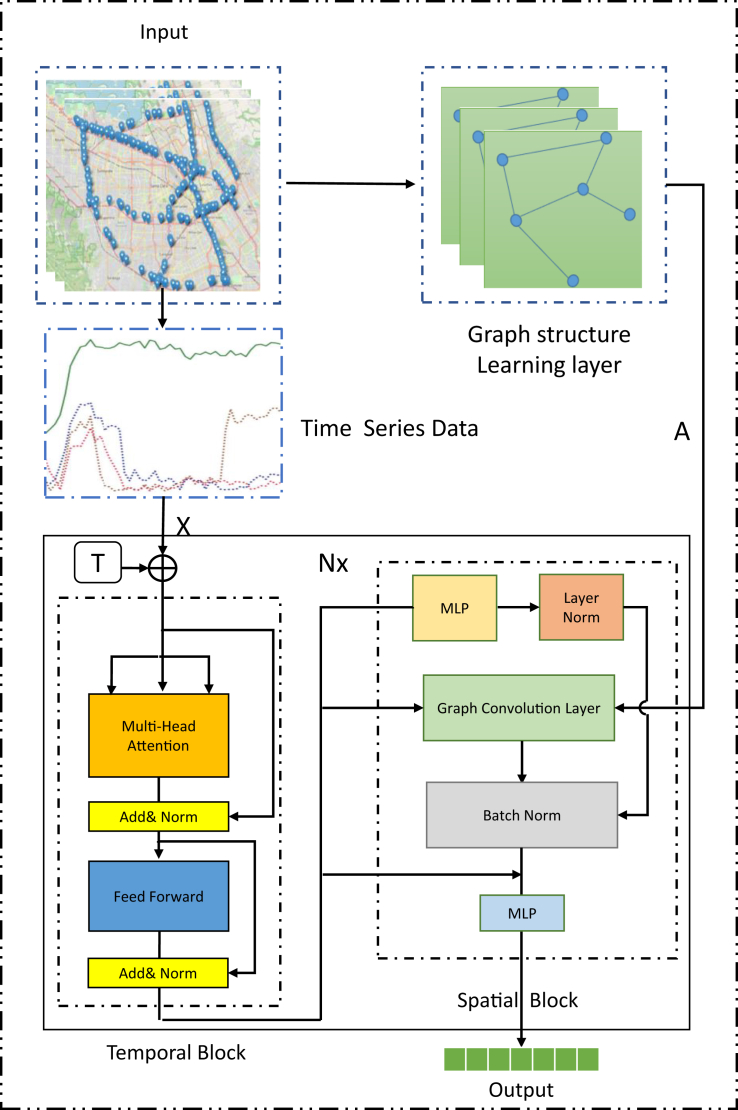
The overall framework of TSTGNN

Our proposed model in Figure 2 primarily employs temporal transformer layers to manage long-term temporal dependencies, utilizing a self-attention mechanism to achieve a global comparison method that measures the impact between time nodes. The self-attention mechanism allows the model to weigh the relationships among different time steps and extract relevant features from the input sequence, thereby capturing complex temporal patterns more effectively than traditional recurrent networks.

In contrast to methods that rely on fixed graph structures, our approach considers both fixed graph structure learning methods and graph structure learning methods for graph convolution layers. Moreover, our graph convolution layers consist of two primary components: a graph structure learning module and a graph convolution operation. The graph structure learning module is responsible for learning the adaptive graph structures in each graph layer, while the graph convolution operation propagates and aggregates information across adjacent nodes in the learned graph structure. By combining these components, our model can effectively capture and represent the dynamic spatial dependencies in geographical traffic networks.

To handle the complicated spatial dependency, we introduce a layer normalization module and graph convolution layers that capture the spatial structure of geographical traffic networks. The layer normalization method between different layers allows the model to adjust and refine the information representations iteratively, leading to improved heterogeneous spatial modeling as the information flows through the network. This adaptability enables the model to generalize well to various geographical traffic datasets and scenarios.

In summary, our novel deep transformer-based spatiotemporal graph learning model first leverages transformer layers with self-attention mechanisms to handle long-term temporal dependencies, and then proposes adaptive graph convolution layers to capture dynamic spatial dependencies and a layer normalization approach assist to capture the spatiotemporal heterogeneous relationship. This combination of techniques results in a powerful and flexible model, enabling to forecast of geographical traffic in diverse and complex settings effectively. Finally, we select the double transition matrix as the initial graph structure combined with the adaptive adjacency matrix, 8 heads and 10 layers for the transformer, 32 residual channels for graph convolution, and 512 for the end channel of multilayer perceptron (MLP) output. Moreover, the more detailed formulations and equations can be seen in STAR methods.

### Baseline

In this section, we provide a brief overview of the baseline methods (in total, we use 18 baseline models) used for comparison with our proposed deep transformer-based spatiotemporal graph learning model for geographical traffic forecasting. The selected baselines include several state-of-the-art methods, as well as traditional techniques for time series forecasting. The partial baselines are as follows.

(1) ARIMA: the autoregressive integrated moving average (ARIMA) model is a traditional statistical method for time series forecasting. ARIMA models the future values of a time series as a linear combination of past values and past error terms. The model is particularly suitable for univariate time series data with a linear trend and no seasonal components.
(2) STGCN: the spatiotemporal graph convolutional network (STGCN) is a CNN designed for spatiotemporal data on graphs. The model consists of two types of graph convolution operations, one for spatial dependencies and the other for temporal dependencies. STGCN uses a Chebyshev polynomial-based graph convolution layer for spatial feature extraction and a 1D convolution layer for temporal feature extraction.
(3) DCRNN: the diffusion convolutional recurrent neural network (DCRNN) is a model that combines graph convolution operations with RNNs to capture both spatial and temporal dependencies in geographical traffic data. The model employs a bidirectional random walk-based graph diffusion process to define the graph convolution operation, and it uses a sequence-to-sequence learning framework with an encoder-decoder architecture for geographical traffic forecasting.
(4) Graph WaveNet: Graph WaveNet is a deep learning model that combines GCNs and gated recurrent units (GRUs) with the WaveNet architecture for geographical traffic forecasting. The model captures spatial dependencies through the use of a graph convolution layer and models temporal dependencies with a dilated causal convolution layer. It also employs an adaptive dependency matrix to capture dynamic spatial dependencies and a data-driven graph construction method for better graph learning.
(5) STGODE: in addressing the challenge of modeling complex spatiotemporal correlations in traffic flow prediction, STGODE, utilizing deep networks and semantical connections for superior performance by implementation semantical adjacency matrix and well-design temporal dilated convolution structure.
(6) StemGNN: the spectral temporal graph neural network (StemGNN) improves multivariate time-series forecasting accuracy by jointly capturing inter-series correlations and temporal dependencies in the spectral domain. It combines graph Fourier transform and discrete Fourier transform in an end-to-end framework, learning inter-series correlations automatically.
(7) STG-NCDE^34^: STG-NCDE is designed for spatiotemporal geographical traffic forecasting. The method combines neural controlled differential equations (NCDEs) for temporal and spatial processing. NCDEs are a breakthrough concept for processing sequential data. The proposed method is robust to missing observations, making it suitable for real-world environments where sensing values can be missing.

### Evaluation metrics and comparison

**Figure 3:**
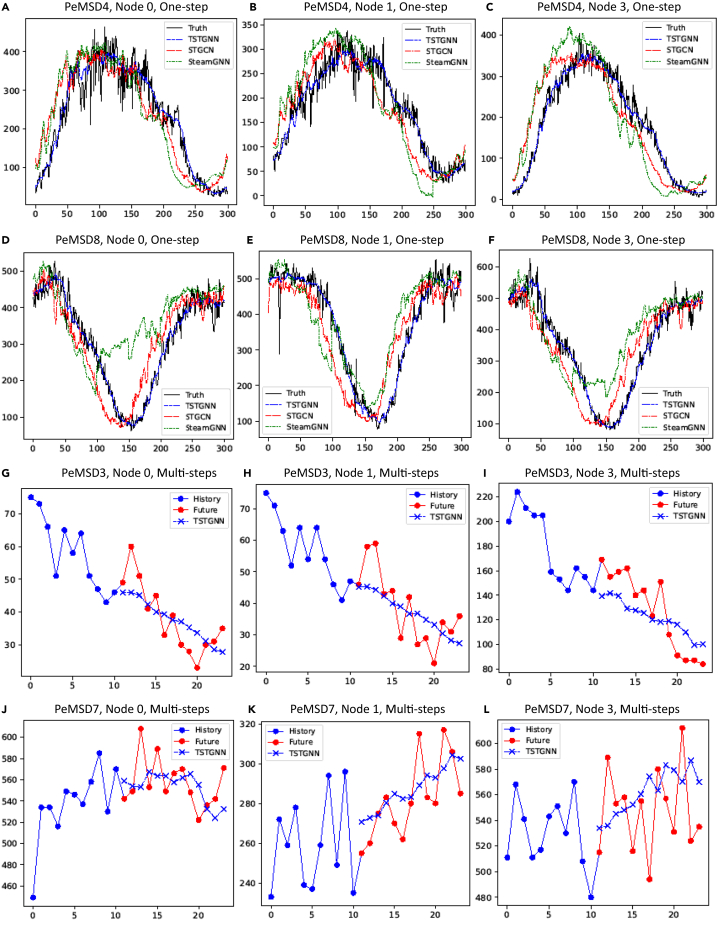
One-step and multi-step visualization Visualization of results, (A–C) are one-step forecasting of nodes 0, 1, and 3 in PeMSD4, horizontal axis is time and the vertical axis is volume prediction as the same to the PeMSD8 in (D–F). (G–I) are the multi-steps forecasting of nodes 0, 1, and 3 in PeMSD3, horizontal axis is time and the vertical axis is volume prediction as the same to the PeMSD7 in (J–L).

The datasets are already separated 6:2:2 into training, validating, and testing sets. Five minutes separate two consecutive time points in these datasets. After examining the previous 12 graph snapshots, i.e., *N* = 11, all existing works, including ours, use the prediction parameters T = 12, L = 1 to gradually display our prediction results using the window as can be seen in Figures 3A–3F. Note that the index of the snapshot i of the graph starts at 0. In conclusion, we do a 12-sequence-to-12-sequence forecast in Figures 3G–3L, which is the standard in this paper.

We define the mean absolute error (MAE), the root-mean-square error (RMSE) and the mean absolute percentage error (MAPE) as the evaluation matrix for our different baselines.

(Equation 1) $$\text{MAE}=\frac{1}{N}\sum_{i=1}^{N}\left|{\hat{y}}_{i}-y_{i}\right|\text{,}$$

(Equation 2) $$\text{RMSE}=\sqrt{\frac{1}{N}\sum_{i=1}^{N}{\left({\hat{y}}_{i}-y_{i}\right)}^{2},}$$

(Equation 3) $$\text{MAPE}=\frac{1}{N}\sum_{i=1}^{N}\left|\frac{{\hat{y}}_{i}-y_{i}}{{\hat{y}}_{i}}\right|\times 100\%\text{,}$$

In summary, observing the results in Table 2, it is obvious that our method has achieved state-of-the-art beyond most existing methods, ranging from traditional time series modeling to methods based on deep learning and spatial temporal graphs. Through the visualization results in Figures 3A–3F, it can also be seen that our method has well fitted the trend of data compared in one-step with the selected baseline including STGCN and StemGNN. In a more detailed presentation, our model has also achieved prediction ability in the multi-step prediction range as can be seen in Figures 3G–3L.

**Table 2:** Forecasting error on PeMSD3, PeMSD4, PeMSD7, and PeMSD8

Model	PeMSD3			PeMSD4			PeMSD7			PeMSD8		
	MAE	RMSE	MAPE	MAE	RMSE	MAPE	MAE	RMSE	MAPE	MAE	RMSE	MAPE
HA	31.58	52.39	33.78%	38.03	59.24	27.88%	45.12	65.64	24.51%	34.86	59.24	27.88%
ARIMA	35.41	47.59	33.78%	33.73	48.80	24.18%	38.17	59.27	19.46%	31.09	44.32	22.73%
VAR^35^	23.65	38.26	24.51%	24.54	38.61	17.24%	50.22	75.63	32.22%	19.19	29.81	13.10%
GRU-ED	19.12	32.85	19.31%	23.68	39.27	16.44%	27.66	43.49	12.20%	22.00	36.22	13.33%
DSANet^36^	21.29	34.55	23.21%	22.79	35.77	16.03%	31.36	49.11	14.43%	17.14	26.96	11.32%
STGCN	17.55	30.42	17.34%	21.16	34.89	13.83%	25.33	39.34	11.21%	17.50	27.09	11.29%
DCRNN	17.99	30.31	18.34%	21.22	33.44	14.17%	25.22	38.61	11.82%	16.82	26.36	10.92%
Graph WaveNet	19.12	32.77	18.89%	24.89	39.66	17.29%	26.39	41.50	11.97%	18.28	30.05	12.15%
ASTGCN	17.34	29.56	17.21%	22.93	35.22	16.56%	24.01	37.87	10.73%	18.25	28.06	11.64%
STG2Seq	19.03	29.83	21.55%	25.20	38.48	18.77%	32.77	47.16	20.16%	20.17	30.71	17.32%
LSGCN	17.94	29.85	16.98%	21.53	33.86	13.18%	27.31	41.46	11.98%	17.73	26.76	11.20%
STSGCN	17.48	29.21	16.78%	21.19	33.65	13.90%	24.26	39.03	10.21%	17.13	26.80	10.96%
AGCRN	15.98	28.25	15.23%	19.83	32.26	12.97%	22.37	36.55	9.12%	15.95	25.22	10.09%
STFGNN	16.77	28.34	16.30%	20.48	32.51	16.77%	23.46	36.60	9.21%	16.94	26.25	10.60%
STGODE	16.50	27.84	16.69%	20.84	32.82	13.77%	22.59	37.54	10.14%	16.81	25.97	10.62%
Z-GCNETs	16.64	28.15	16.39%	19.50	31.61	12.78%	21.77	35.17	9.25%	15.76	25.11	10.01%
StemGNN	15.98	27.12	16.30%	22.40	35.33	15.47%	22.89	37.32	9.53%	16.66	26.06	11.57%
STG-NCDE	$\underline{15.57}$	$\underline{27.09}$	$\underline{15.06}\%$	$\underline{19.21}$	$\underline{31.09}$	$\underline{12.76}\%$	$\underline{20.53}$	$\underline{33.84}$	8.80%	**15.45**	$\underline{24.81}$	9.92%
Ours	**15.51**	**26.78**	15.02%	**19.06**	**30.52**	12.72%	**20.40**	**33.83**	8.72%	$\underline{15.69}$	**24.72**	9.86%

Bold text indicates the best results in all methods.

## Discussion

We further explore the error, interpretability, and reliability of traffic prediction under maps in real scenarios through this model. This includes spatiotemporal heterogeneous relationship, error visualization on maps, and multi-step prediction in time dimensions.

### Spatiotemporal heterogeneous relationship

In the scholarly discourse on spatiotemporal graphs, the concept of heterogeneity is interpreted and analyzed through various lenses. For instance, ASTGNN primarily addresses the heterogeneity manifest in the range of values across spatial dimensions. In contrast, ST-WA extends this analysis to include the heterogeneity associated with long-term temporal variations. MegaCRN^37^ delves into the heterogeneity induced by the interplay of road configurations and temporal dynamics. Meanwhile, ST-SSL^38^ spatio concentrates on the disparities in spatial distributions and temporal patterns. Our research endeavors to explore a distinct aspect of heterogeneity: the variability in the long-term feature changes (such as the rate of traffic flow change and series trend). This focus is pivotal because, in constructing graph structures, there is a common reliance on the patterns of similarity based on distance or current flow metrics. Such an approach, however, does not adequately account for the specific impacts of rate of long-term feature change heterogeneity on the information structure.

To address this gap, our methodology integrates a comprehensive framework that combines the strengths of fixed graphs, adaptive graphs, and layer normalization. This tripartite approach is designed to capture implicitly the nuanced effects of rate change heterogeneity, thereby enriching our understanding and modeling of spatiotemporal dynamics in traffic flows. This innovative perspective offers a more robust and nuanced understanding of heterogeneity, paving the way for more precise and effective graph-based analyses in the realm of traffic forecasting and beyond.

**Figure 4:**
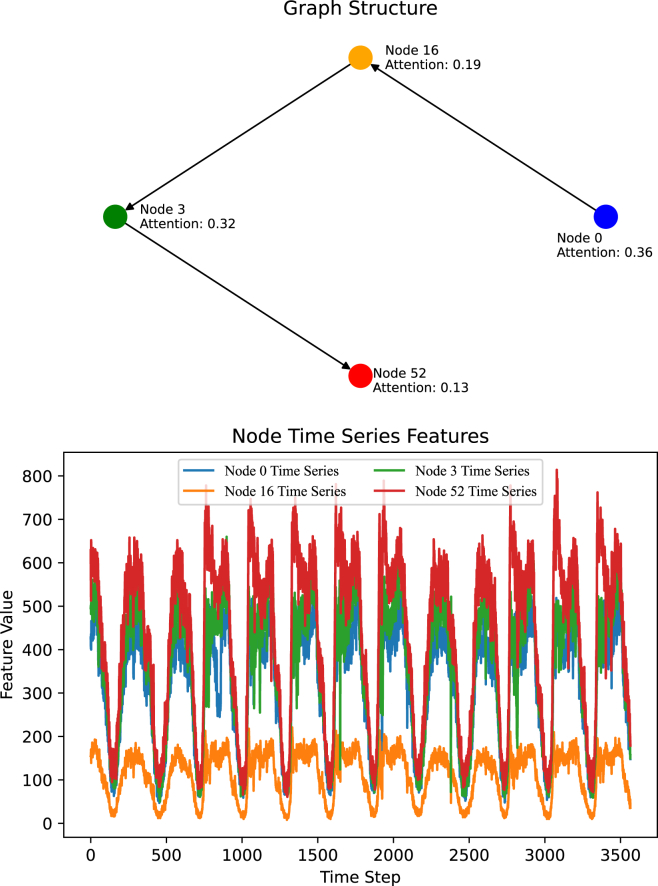
Visualization of the spatial graph structure and temporal time series for nodes 0, 16, 3, and 52 The attention score is calculated by TSTGNN based on the spatiotemporal relationship of each node to node 0.

To analyze the spatiotemporal heterogeneous relationships among nodes, we visualize their spatial graph structure and temporal time series in Figure 4. From the figure, we can observe that node 0 and node 16 are directly connected in the graph, which are close in spatial distance. However, their time series are dissimilar as shown in the bottom of Figure 4. Therefore, we should not pay attention to node 16, when we try to model the temporal patterns of node 0. Even, node 3 is the two-hop neighbor of node 0, its time series is highly similar to node 0. We should give it higher attention to capture the relatedness. For node 52, despite its temporal patterns being similar to node 0, its importance to node 0 is small due to the long spatial distance.

From the observation, it is essential to model the spatiotemporal relation between nodes for time series forecasting. TSTGNN that simultaneously considers the graph structure and temporal information successfully captures such relationship between nodes. This is evident by the attention score calculated by the TSTGNN. For node 0, TSTGNN gives the highest score since we try to forecast the traffic for it. The node 3 gets the second-highest score due to the spatiotemporal relationship. Meanwhile, node 16 and node 52 have been assigned a small weight based on their relatedness.

### Visualization of error

**Figure 5:**
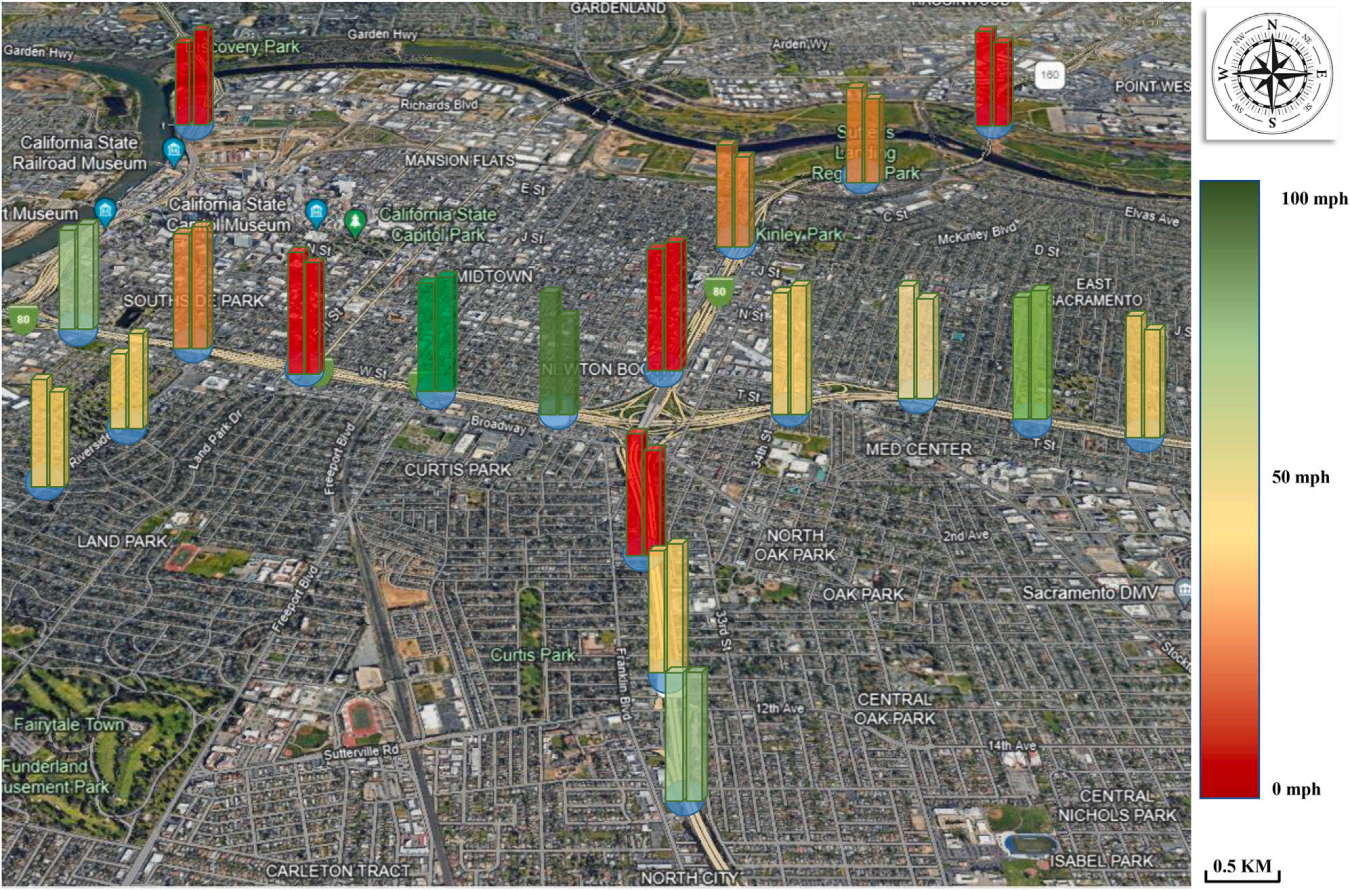
Traffic flow in city of Sacramento, each pair of panels represents the observed value of the traffic flow at a monitoring point Color changes are based on different speeds.

We collected data from key roadways some partial speed monitoring points within the city of Sacramento using PeMSD3 and created visualizations that showcased both the raw data and our predicted data in Figure 5. The raw data are presented on the left, while the predicted data are displayed on the right. Upon comparing the two sets of data, we discovered that our margin of error was minimal, enabling us to accurately capture speed fluctuations across the entire area and quantifying future development trends.

The visualized graph further illuminates the spatiotemporal mismatch relationship we have previously discussed. Geographically, adjacent nodes may not consistently exhibit the same speeds. For instance, when traveling from west to east on the highway, there is a noteworthy speed difference between the third and fourth nodes (which are adjacent), but the speed variance between the sixth and seventh nodes (also adjacent) is minimal. Interestingly, the speed difference between the sixth and ninth nodes (which are not adjacent) is also quite small, corroborating our perspective of spatiotemporal heterogeneous relationships.

### Visualization of the one-step and multi-steps prediction

We assessed the predictive capabilities of our model for both one-step and multi-step forecasting. We selected node 0, node 1, and node 3 of PeMSD4 and PeMSD8 for one-step prediction visualization. Visualization of multi-step predictions was performed on node 0, node 1, and node 3 of PeMSD3 and PeMSD7.

Furthermore, in the case of one-step predictions, it is evident that our model outperforms other baseline models, demonstrating a superior fit to the original data and effective forecasting. When there are significant fluctuations in the data, our model output remains robust without any deviation. However, other models will have certain offsets and distortions. For example, In PeMSD8 node 0, StemGNN has undergone a significant shift. The results of STGCN always seem to lag behind the original data for a period of time in all of datasets. In the realm of multi-step predictions, our model also exhibits proficiency in predicting future trends.

### Conclusion

In conclusion, this study presents a novel deep transformer-based spatial-temporal graph (TSTGNN) learning model for geographical traffic forecasting, addressing the challenges faced by traditional and graph-based deep learning methods in capturing long-term temporal dependencies and modeling dynamic spatial dependencies. Our model leverages temporal transformers to effectively capture long-term temporal patterns in traffic data without resorting to data fusion. Additionally, we introduce adaptive graph structure across different graph convolution layers, enabling the model to capture dynamic spatial dependencies and implement layer normalization to handle heterogeneous relationships, and adapt to diverse traffic scenarios.

We have extensively evaluated our model on four primary traffic datasets (PeMSD3, PeMSD4, PeMSD7, and PeMSD8), demonstrating state-of-the-art results in comparison to existing methods. Furthermore, we conducted ablation studies and parameter sensitivity analyses to validate the robustness and generalizability of our approach.

Our model offers a promising and efficient solution for accurate traffic forecasting, contributing to the advancement of the state of the art in this critical field of study. With the continuous evolution of urban transportation planning, traffic management, and intelligent transportation systems, our model stands poised to play a pivotal role in empowering policymakers to make informed decisions, ultimately leading to the optimization of efficiency and safety on highways.

### Limitations of the study

Although our model has been fairly compared with existing models on public test datasets and has achieved the state of the art, there is still a possibility for our results to be further improved and tested on more reliable real-world data. Although our model has initially achieved our goals, its computational complexity could be still further optimized in future work.

## STAR★Methods

### Key resources table

**Table:** 

REAGENT or RESOURCE	SOURCE	IDENTIFIER
**Deposited data**		

PeMD3	Datasets are generated from Performance Measurement System (PeMS) Data	https://pems.dot.ca.gov/
PeMD4	Datasets are generated from Performance Measurement System (PeMS) Data	https://pems.dot.ca.gov/
PeMD7	Datasets are generated from Performance Measurement System (PeMS) Data	https://pems.dot.ca.gov/
PeMD8	Datasets are generated from Performance Measurement System (PeMS) Data	https://pems.dot.ca.gov/

**Software and algorithms**		

BasicTS	A Standard and Fair Time Series Forecasting Benchmark and Toolkit.	https://github.com/zezhishao/BasicTS/tree/master

### Resource availability

#### Lead contact

Further information and requests for resources and reagents should be directed to and will be fulfilled by the lead contact, Shirui Pan (s.pan@griffith.edu.au).

#### Materials availability

This study did not generate new datasets.

#### Data and code availability

• Datasets are generated from PeMS Data Source (https://pems.dot.ca.gov/) and we implement our model based on BasicTS (https://github.com/zezhishao/BasicTS/tree/master).
• All original code has been deposited at Github (https://github.com/RManLuo/TSTGNN.git) and is publicly available as of the date of publication. DOIs are listed in the key resources table.
• Any additional information required to reanalyze the data reported in this paper is available from the lead contact upon request.

### Method details

This section presents our proposed deep transformer-based spatiotemporal graph learning model for geographical traffic forecasting. The methodology comprises two main components: (1) the temporal transformer to capture time dependencies and (2) the GNN with adaptive normalization to handle spatial dependencies.

#### Problem definition

In this section, we formally define the geographical traffic forecasting problem and the corresponding loss function. Consider a geographical traffic sensor network represented as a graph G=(V,E), where V denotes the set of vertices (sensors), and E represents the set of edges (connections between sensors). The traffic forecasting problem can be stated as follows: Given a sequence of historical traffic data $X_{T}=x_{1},x_{2},\ldots ,x_{T}$, where $x_{t}\in R^{N\times D}$ is the geographical traffic feature matrix at time step t, the objective is to forecast the geographical traffic feature matrix $X_{T+L}$ at a future time step T+L, where L is the forecasting length $\left(x_{t+1},x_{t+2},\ldots ,x_{T+L}\right)$. The input to the model is a tensor $X\in R^{N\times T\times D}$, where N indicates the number of nodes (sensors), T represents the length of the input sequence, and D corresponds to the number of features. The model’s output is a tensor $\hat{X}\in R^{N\times L\times D}$, signifying the predicted geographical traffic feature matrices for each batch and node. To evaluate the performance of the traffic forecasting model, we employ the MAE ( MAE ) as the loss function. The MAE measures the average absolute difference between the predicted geographical traffic feature matrices $\hat{X}$ and the ground-truth traffic feature matrices $\in R^{N\times L\times D}$ :

$$\text{MAE}\;\left(\hat{X},X\right)=\frac{1}{N\times L\times D}\sum_{n=1}^{N}\;\sum_{l=1}^{L}\;\sum_{d=1}^{D}\;\left({\hat{X}}_{n,l,d}-X_{n,l,d}\right)$$

Here, ${\hat{X}}_{n,l,d}$ and $X_{n,l,d}$ represent the predicted and ground-truth values, respectively, for the n-th node, l-th time step, and d-th feature dimension. The traffic forecasting model aims to minimize the MAE loss, motivating the model to generate accurate predictions that closely match the ground-truth traffic data. By optimizing the model using the MSE loss, we ensure that our deep transformer-based spatiotemporal graph learning model can provide reliable and accurate traffic forecasts for various geographical traffic datasets. The summary of notation has been attached in Table S1 in Supplemental.

#### Temporal transformer layers

The temporal transformer is based on the transformer architecture proposed by Vaswani et al.^33^ It consists of a multi-head self-attention mechanism, which is utilized to model the long-term temporal dependencies in traffic data denoted as X. The multi-head self-attention mechanism computes the attention weights between each pair of time steps in the input sequence. For each attention head h, the self-attention weights are calculated as follows:

$$A_{h}=\text{softmax}\;\left(\frac{Q_{h}K_{h}^{T}}{\sqrt{d_{k}}}\right)\text{.}$$

where $Q_{h},K_{h}$, and $V_{h}$ are the query, key, and value matrices, respectively, obtained by linearly projecting the input sequence using learnable weight matrices $W_{h}^{Q}\in R^{D\times d_{k}},W_{h}^{K}\in R^{D\times d_{k}}$, and $W_{h}^{V}\in R^{D\times d_{v}}\text{:}$

$$\begin{array}{r} Q_{h}={\text{XW}}_{h}^{Q}\text{,}\\ K_{h}={\text{XW}}_{h}^{K}\text{,}\\ V_{h}={\text{XW}}_{h}^{V}\text{.} \end{array}$$

The self-attention output for each head is computed as the weighted sum of the value matrices:

$$O_{h}=A_{h}V_{h}\text{.}$$

The outputs of all attention heads are concatenated and linearly projected to obtain the final multi-head self-attention output:

$$O=\text{Concat}\;\left(O_{1},\ldots ,O_{H}\right)W^{O}\text{.}$$

where $W^{O}\in R^{Hd_{v}\times D}$ is a learnable weight matrix. The temporal transformer layers also include position-wise feedforward networks (FFNs) to process the self-attention output further. The FFNs consist of two linear layers with a ReLU activation function in between:

$$F\left(O\right)=\text{ReLU}\;\left(OW^{1}+b^{1}\right)W^{2}+b^{2}\text{.}$$

where $W^{1}\in R^{D\times d_{ff}},W^{2}\in R^{d_{ff}\times D},b^{1}\in R^{d_{ff}}$, and $b^{2}\in R^{D}$ are learnable weight and bias parameters, respectively. The output of the temporal transformer layers, denoted as $Z\in R^{N\times L\times D}$, is obtained by applying layer normalization and residual connections:

Z=LayerNorm(X+F(LayerNorm(O))).

#### Layer normalization for dynamic relationships

Inspiring by the graph-free spatial (GFS) learning module,^39^ we equip layer normalization with a linear projection and ReLU activation function in order to extract spatial correlations between vertices. Considering that residual connection increases the representation ability of graph convolution, we employ a residual connection in GFS learning to increase the representation ability of layer normalization. The expression for the calculation of layer normalization is:

$$\mathrm{LN}\left(V\right)=\left[\frac{\text{Diag}\;\left(W\right)}{\sigma_{V}}\left(I-N\right)\right]V\text{.}$$

where Diag(W) is a diagonal matrix whose diagonal element in each row corresponds to the element in the corresponding row of $W,\sigma_{V}=\text{Var}\;\left[V\right]$ represents the standard deviation of $V\in R^{N\times 1}$, and $N\in R^{N\times N}$ denotes a matrix filled with $\frac{1}{N}$. The $\frac{\text{Diag}\;\left(W\right)}{\sigma_{V}}\left(I-N\right)$ can be regarded as an adjacency matrix A measuring the relationship between nodes. Regarding the calculation of layer normalization, the diagonal matrix Diag(W) ensures the diversity among the values in different rows in A, and A contains data-driven term $\sigma_{V}$ and learnable parameters W, which determine that layer normalization is effective and flexible in aggregating vertices. In case the dimensionality of the feature is D, we should calculate each dimension individually with the method mentioned above. Specifically, given input $Z\in R^{L\times N\times d_{in}}$ from the temporal transformer layer, GFS first applies a linear projection and a ReLU activation function on the feature dimension, i.e.,

$$Z^{\prime }=\text{ReLU}\;\left({\text{ZW}}_{s1}+b_{s1}\right)\text{.}$$

where $W_{s1}\in R^{d_{in}\times d_{\text{out}\;}}$ and $b_{s1}\in R^{d_{\text{out}\;}}$ are learnable parameters, and $Z^{\prime }$ is the result of projection. Next, a layer normalization is applied on $V^{\prime }$ on both vertex and feature dimensions, i.e.,

$$\hat{Z}=\frac{Z^{\prime }-E\left[Z^{\prime }\right]}{\sqrt{\text{Var}\;\left[Z^{\prime }\right]}}W_{s2}+B_{s2}\text{.}$$

where $W_{s2}\in R^{N\times d_{\text{out}\;}}$ and $B_{s2}\in R^{N\times d_{\text{out}\;}}$ are learnable affine parameters, and $\hat{Z}$ is the output of layer normalization. To generate the final output $Z_{\text{out}\;}\in R^{L\times N\times d_{\text{out}\;}}$ of GFS, a residual connection integrated with linear projection is applied on the input and added to $\hat{Z}$, i.e.,

$$\begin{array}{c} Z_{\text{res}\;}=\text{ReLU}\;\left(ZW_{\text{res}\;}+b_{\text{res}\;}\right)\text{.}\\ Z_{\text{out}\;}=Z_{\text{res}\;}+\hat{Z}\text{.} \end{array}$$

where $W_{\text{res}\;}\in R^{d_{\text{in}\;}\times d_{\text{out}\;}}$ and $b_{\text{res}\;}\in R^{d_{\text{out}\;}}$ are learned parameters.

#### Graph convolution layers

To model spatial dependencies in traffic sensor data, we utilize a GNN with an adaptive normalization method. The traffic sensor network is represented as a graph G=(V,E), where V is the set of vertices (sensors) and E is the set of edges (connections between sensors). The input to the GNN is the output of the temporal transformer, denoted as $Z\in R^{N\times L\times D}$. The GNN consists of a series of graph convolution layers that capture spatial dependencies in the traffic data. In each layer, the adaptive normalization method adjusts the graph structure based on the feature representations at the current layer. Graph convolution is a critical operation that extracts a node’s features given its structural information. Our approach is inspired by the graph wavenet [10]. Its advantages are that it is a compositional layer, its filter is localized in space, and it supports multi-dimensional inputs. Let $\tilde{A}\in R^{N\times N}$ denote the normalized adjacency matrix with self-loops, $Z\in R^{N\times D}$ denote the input signals after the previous operations, $H\in R^{N\times M}$ denote the output, and $W\in R^{D\times M}$ denote the model parameter matrix. In Kipf and Welling [44], the graph convolution layer is defined as follows:

$$H=\tilde{A}\text{ZW.}$$

Typically, the original adjacency matrix or the identity matrix is utilized. In contrast, Li et al. [9] proposed a diffusion convolution layer that is effective in spatiotemporal modeling. They modeled the process of graph signal diffusion with K discrete steps. We generalize its diffusion convolution layer into the form of the following equation:

$$H^{\left(l+1\right)}=\sigma \left(\hat{A}H^{\left(l\right)}W^{\left(l\right)}\right)\text{.}$$

where feature representation at layer l is denoted as $H^{\left(l\right)}$, and $W^{\left(l\right)}$ is the learnable weight matrix for layer l. An activation function, such as the ReLU function, is applied and denoted as σ. By utilizing the adaptive normalization method, the GNN can effectively capture dynamic spatial dependencies and adapt to various traffic scenarios. Fixed Graph Structure Matrix: The diffusion convolution layer can be generalized into the form of Equation 18,^10^ as:

$$H=\sum_{k=0}^{K}\;P^{k}ZW_{k}\text{.}$$

where the power series of the double transition matrix is represented by $P^{k}$. In the case of an undirected graph, the double transition matrix is defined as P=A/ rowsum(A). For a directed graph, the diffusion process has two directions: the forward and backward directions. The forward transition matrix is defined as $P_{f}=A/$ rowsum (A), and the backward transition matrix is defined as $P_{b}=A^{T}/$ rowsum $\left(A^{T}\right)$. By utilizing the forward and backward double transition matrices, the diffusion graph convolution layer can be expressed as:

$$H=\sum_{k=0}^{K}\;P_{f}^{k}ZW_{k1}+P_{b}^{k}ZW_{k2}\text{.}$$

Self-adaptive Graph Structure Matrix: To learn hidden spatial dependencies without prior knowledge, we utilize a self-adaptive adjacency matrix^10^ denoted as ${\tilde{A}}_{adp}$. This matrix is learned end-to-end through stochastic gradient descent, enabling the model to discover spatial dependencies on its own. To accomplish this, we randomly initialize two node embedding dictionaries with learnable parameters $E_{1},E_{2}\in R^{N\times c}$. The self-adaptive adjacency matrix is proposed as follows:

$${\tilde{A}}_{adp}=\text{softmax}\;\left(\text{ReLU}\;\left(E_{1}E_{2}^{T}\right)\right)\text{.}$$

We refer to E1 as the source node embedding and E2 as the target node embedding. By computing the element-wise product of E1 and E2, we obtain the spatial dependency weights between source and target nodes. To eliminate weak connections, we apply the ReLU activation function. Next, we normalize the self-adaptive adjacency matrix using the SoftMax function. The resulting normalized matrix can be viewed as the double transition matrix of a hidden diffusion process. By combining pre-defined spatial dependencies and self-learned hidden graph dependencies, we propose the following graph convolution layer:

$$H=\sum_{k=0}^{K}\;P_{f}^{k}ZW_{k1}+P_{b}^{k}ZW_{k2}+{\tilde{A}}_{apt}^{k}ZW_{k3}\text{.}$$

When the graph structure is unavailable, we propose to use the self-adaptive adjacency matrix alone to capture hidden spatial dependencies, i.e.,

$$H=\sum_{k=0}^{K}\;{\tilde{A}}_{apt}^{k}ZW_{k}\text{.}$$

When the graph structure is unavailable, we propose to use the self-adaptive adjacency matrix alone to capture hidden spatial dependencies, i.e.,

$$H=\sum_{k=0}^{K}\;{\tilde{A}}_{adp}^{k}ZW_{k}\text{.}$$

#### Computation complexity

In this section, we analyze the computation complexity of the proposed method. Given an input time series $X\in R^{N\times L\times D}$, the complexity of a single temporal transformer layer is $O\left(LN^{2}H\right)$, the complexity of the graph convolution layer is $O\left(ND^{2}\right)$, and the complexity of the Layer Normalization is O(ND). By staking n layers, the overall complexity is calculated as $O\left(n\left(LN^{2}H+ND^{2}+ND\right)\right)$.

#### Computing infrastructures

Our proposed learning framework is implemented base on the BasicTS: A Time Series Benchmark and Toolkit. All experiments were conducted on a computer equipped with NVIDIA RTX 2080Ti GPU and an Intel Core i9-10900X (3.70 GHz) CPU and 32 GB of RAM. Finally, we determine the epoch 100, batch size 32.

#### Data preprocessing

We annotate the time series in a time increasing manner and add it to the features to preserve the sequence information. Secondly, we construct the graph link matrix based on the distance between traffic measurement points.^40^ In the end, we adopted a *Z* Score approach to normalize and de normalize our features.^41^

#### Ablation study

We construct five variants of our method to study the effectiveness of core components. Specifically, w\ o TR: TSTGNN disables the temporal transformer function, w\o ADP: TSTGNN removes the self-adaptive graph structure matrix w\OR: TSTGNN utilizes the original adjacency matrix, w\ID: TSTGNN utilize the identity matrix.

The experimental results are in Figure S1 in Supplemental, where our full model that utilizes the transition adjacency matrix equipped with all components has the best performance across the dataset.

In summary, by analyzing the impact of various modules on the final findings, we can determine that the temporary transformer layer has the most impact on various data in our method, showing that temporary dependency has a substantial impact on the forecasting framework. Despite the fact that different spatial modules have distinct effects, they can significantly improve results. In comparison to conventional approaches of aggregating temporal data in Table 2, the addition of spatial modules can significantly improve the performance. We can find some observation.

(1) First, Observing the effect of different modules on PeMSD3 in Figure S1A makes it easy to determine that when the temporal transformer layers is removed, the indicators of *MAE*, *RMSE*, and *MAPE* will decrease by almost 13%, 18%, and 4%, respectively, compared to our full model. Removing the self-adaptive graph structure matrix has the second-greatest impact, whereas other graph structure methods have a lesser impact. Nevertheless, based on the results, the double transition graph structure transition is the most effective.
(2) Second, as shown in Figure S1B, comparing the full model, it is not difficult to find out that the evaluation matrix will be dropped 11%, 12%, and 15% respectively when removing the temporal transformer layers. Similarly, we also find that the self-adaptive graph structure matrix has the second biggest effect on the results. Although we finally select the double transition graph structure, the most effective one is the original matrix.
(3) Third, after comparing different modules of the method in Figure S1C, we also find the same roles, which is that performance could be decreased by removing the temporal transformer layers about the 18%, 17%, and 17%, among the MAE, RMSE, and MAPE respectively, while other spatial operations have almost the same effect on the performance.
(4) Fourth, in Figure S1D comparing PeMSD3, PeMSD4, and PeMSD7, the temporal transformer layer has the greatest impact on the final results, with a decrease of 20%, 18%, and 17% in MAE, RMSE, and MAPE, while the other layers have nearly the same effect.
(5) Finally, as shown in Figure S2 in Supplemental, we observe the relationship between nodes dynamically changing through layers. We illustrate the relationship between node 0 and nodes 0–10 learned by the layer normalization at each layer. The relationship is closer when the color is lighter. From the results, we can find that the relationships between nodes are adaptively adjusted as their representation transferred between different layers. This enables to better capture their dependencies in the high-dimensional space.

#### Parameter sensitive study

Apart from the experiments on ablation study, we also conduct experiments on other important hyperparameters, including dropout, end channels of MLP output, layers of the temporal transformer, and residual channels of the graph convolution layer to investigate their impacts on our model, as shown in Figure S3 in Supplemental.

In summary, we have comprehensively tested the impact of different hyperparameters on the performance of our model, and the overall results are in line with expectations and laws. The model has not undergone significant changes, although some datasets have some slight fluctuations, which also indicates that our model is relatively robust. Moreover, from the results, each dataset has its own relatively stable optimal value, which indicates our model has generalizability.

We have the following observations.

(1) Dropout is a strategy used to prevent overfitting in neural networks. When training neural networks, dropout shuts off neurons at random, preventing the network from relying heavily on any one neuron for predictions. Thus, the network may be generalized to new data more effectively. Dropout can also reduce redundancy in the neural network, increase training speed, and enhance the network’s generalization capability. We frequently meet noisy or anomalous data in the real world, which can have a substantial impact on the performance of neural networks. Dropout enables neural networks to process these data, hence enhancing the model’s robustness. Within a reasonable range, e.g., from 0.1 to 0.5, moderately increasing the dropout in Figures S3A–S3C, do not have a significant impact on results. However, there will be obvious fluctuations between RMSE and MAPE in PeMSD7 and PeMSD3 respectively.
(2) In the end, MLP output merges temporal and spatial data and maps it to the target space. In general, the number of neurons in the output layer can improve the model’s ability to gather sufficient information in high-dimensional space for learning purposes. However properly increasing the MLP output channels in Figures S3D–S3F does not improve the performance of the model obviously, since the lower dimension of the layers about 32 can adequately capture the information. Thus, Excessive dimensions may increase computational resources and is not favorable to model learning.
(3) Within a reasonable range, e.g., from 2 to 10 in Figures S3G–S3I the performance can be increased and converged as the increasing of the layers of temporal transformer layers, except for the MAPE of the PeMSD3. In general, the more the number of layers a transformer has, the greater its performance. It can extract information relationships and apply them to the calculation of high-dimensional variables more effectively. In other words, the transformer can successfully capture temporal relationships in the temporal dimension and organize them sensibly for representation in a high-dimensional space.
(4) Observing the effect of residual channel in Figures S3J–S3L, we can find that the error curve gradually decreases. This module represents the graph convolution layer’s capacity to obtain spatial data. In general, the larger the dimension, the more information it can acquire, but the more complex its computation. The performance of our model improves as the number of dimensions increases from 8 to 128, except for minor oscillations in PeMSD3.

## Supplemental information

Document S1. Figures S1–S3

Table S1. Summary of the primary notations related to Problem Definition section in STAR Methods
