# Supplementary material for: Deep transformer-based heterogeneous spatiotemporal graph learning for geographical traffic forecasting
Source: iScience. 2024 Jun 25;27(7):110175. doi: 10.1016/j.isci.2024.110175 (PMC11302005; doi:10.1016/j.isci.2024.110175)
Supplement: Document S1. Figures S1–S3 [file mmc1.pdf]

iScience, Volume *27*

Supplemental information

Deep transformer-based heterogeneous spatiotemporal graph learning for geographical traffic forecasting

Guangsi Shi, Linhao Luo, Yongze Song, Jing Li, and Shirui Pan

Supplemental

A PeMSD3 Ablation Study

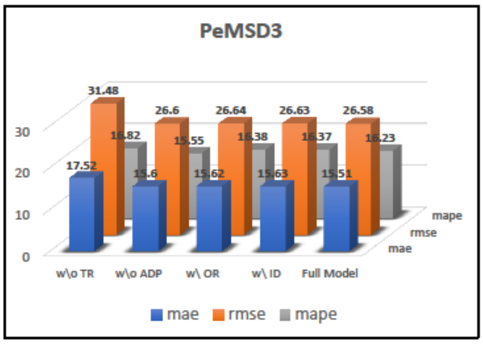

B PeMSD4 Ablation Study

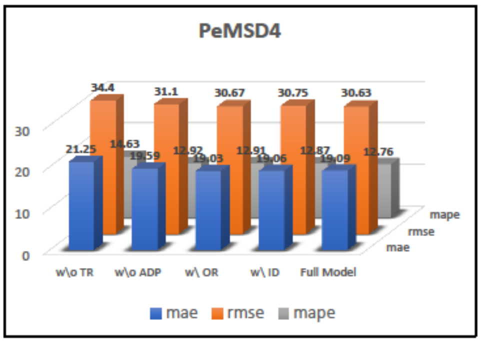

C PeMSD7 Ablation Study

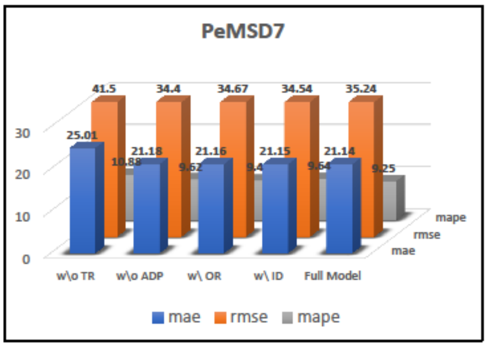

D PeMSD8 Ablation Study

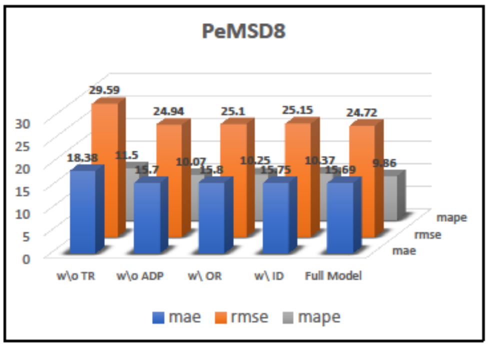

Figure S1: A to D ablation study of TSTGNN in different datasets, which is related to Ablation Study Section in STAR Method

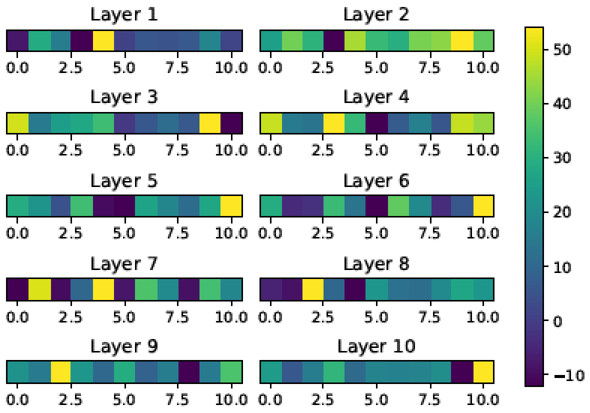

Figure S2: Relationship between node 0 and nodes 0-10 learned by the layer normalization at each layer. The relationship is closer when the color is lighter, which is related to the Ablation Study Section in STAR Method

A Dropout MAE

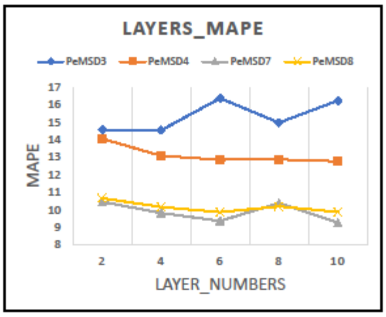

B Dropout RMSE

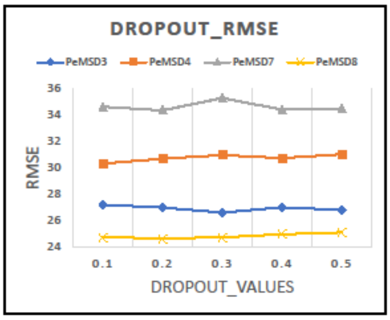

C Dropout MAPE

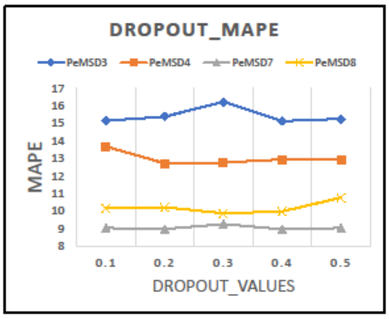

D End Channels MAE

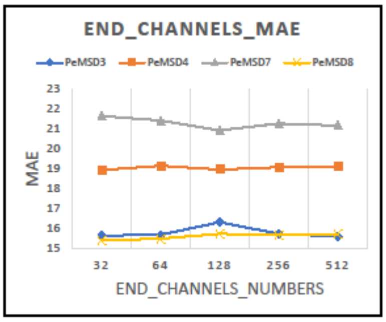

E End Channels RMSE

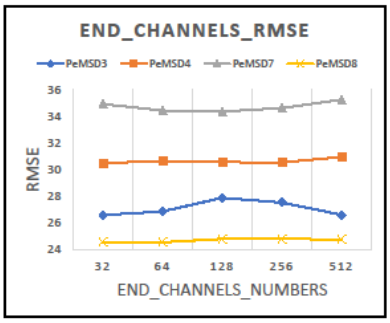

F End Channels MAPE

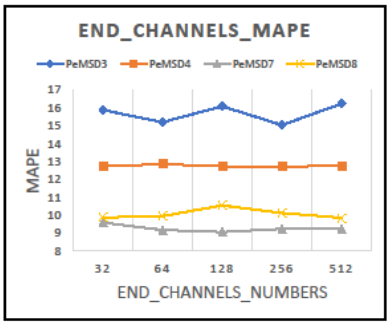

G Layers MAE

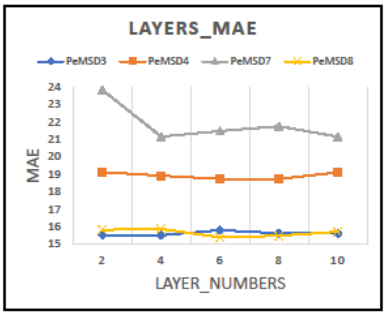

H Layers RMSE

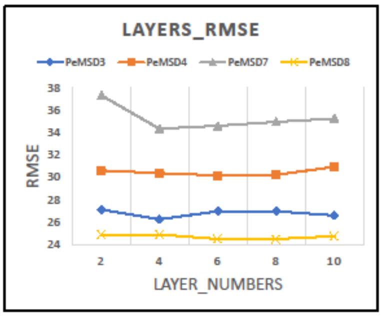

I Layers MAPE

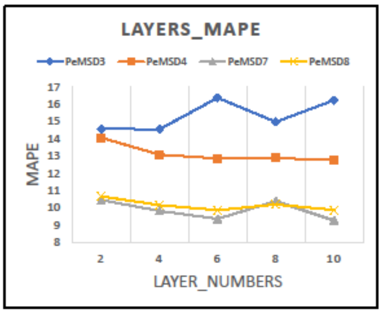

J Residual Channels MAE

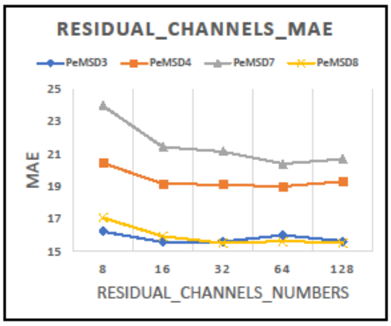

K Residual Channels RMSE

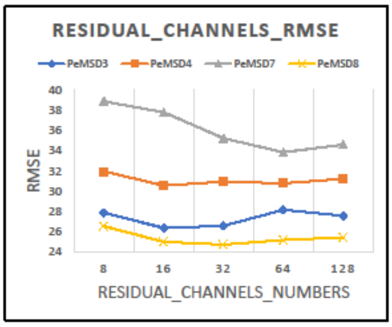

L Residual Channels MAPE

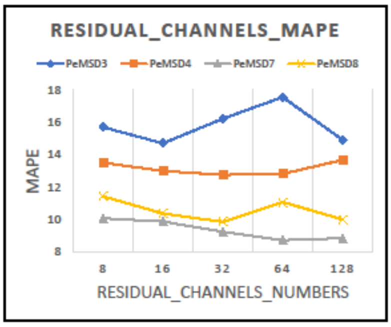

Figure S3: A to L are parameter study of TSTGNN in different datasets, which is related to the Parameter Sensitive Study Section in STAR Method.
