# Supplementary material for: Deep transformer-based heterogeneous spatiotemporal graph learning for geographical traffic forecasting
Source: iScience. 2024 Jun 25;27(7):110175. doi: 10.1016/j.isci.2024.110175 (PMC11302005; doi:10.1016/j.isci.2024.110175)
Supplement: Table S1. Summary of the primary notations related to Problem Definition section in STAR Methods [file mmc2.pdf]

Table S1: Summary of the primary notations related Problem Definition Section in STAR Method.

**Symbols**	**Description**
$\mathcal{G} = (\mathbf{X}, \mathbf{A})$	A graph representing the traffic sensor newtwork
$\mathbf{A} \in \mathbb{R}^{N \times N}$	The adjacency matrix of $\mathcal{G}$
$\mathbf{X} \in \mathbb{R}^{N \times L \times D}$	The node features matrix of $\mathcal{G}$
$\mathbf{Z} \in \mathbb{R}^{N \times L \times D}$	Feature transformation of hidden dimensions
$\mathbf{W}_h^Q \in \mathbb{R}^{D \times d_k}$	Weight matrix for Q of attention
$\mathbf{W}_h^K \in \mathbb{R}^{D \times d_k}$	Weight matrix for K of attention
$\mathbf{W}_h^V \in \mathbb{R}^{D \times d_v}$	Weight matrix for V of attention
$\mathbf{W}_{s2} \in \mathbb{R}^{N \times d_{\text{out}}}$<br>$\mathbf{b}_{\text{res}} \in \mathbb{R}^{N \times d_{\text{out}}}$	Learned parameters for Layer normalization
$\mathbf{W}_{\text{in}} \in \mathbb{R}^{d_{in} \times d_{\text{out}}}$	Learnable weight matrix for input features in GFS module
$\mathbf{W}_{\text{res}} \in \mathbb{R}^{d_{out} \times d_{\text{out}}}$	Learnable residual weight matrix in GFS module
$b_{\text{res}} \in \mathbb{R}^{d_{\text{out}}}$	Learnable residual bias vector in GFS module
$\mathbf{H} \in \mathbb{R}^{N \times D}$	Hidden space of graph convolution layers
$\mathbf{P}^k \in \mathbb{R}^{N \times N}$	Fixed graph structure matrix
$\mathbf{A}_{\text{adaptive}} \in \mathbb{R}^{N \times N}$	Adaptive graph structure matrix
N	The number of nodes in the traffic sensor network graph
D	The dimension of input node feature
T	The number of historical traffic data
H	The number of head of transformer
d_{in}	Input feature dimension in the GFS module
d_{out}	Output feature dimension in the GFS module
